# Nutritional Value of Commercial Protein-Rich Plant Products

**DOI:** 10.1007/s11130-018-0660-7

**Published:** 2018-03-02

**Authors:** Pirjo Mattila, Sari Mäkinen, Merja Eurola, Taina Jalava, Juha-Matti Pihlava, Jarkko Hellström, Anne Pihlanto

**Affiliations:** 1 Natural Resources Institute, 20520 Turku, Finland; 2 Natural Resources Institute, 31600 Jokioinen, Finland

**Keywords:** Faba bean, Lupin, Hemp seed, Buckwheat, Rapeseed press cake, Flaxseed, Quinoa, Nutrients

## Abstract

The goal of this work was to analyze nutritional value of various minimally processed commercial products of plant protein sources such as faba bean (*Vicia faba*), lupin (*Lupinus angustifolius*), rapeseed press cake (*Brassica rapa/napus* subsp. *Oleifera*), flaxseed (*Linum usitatissimum*), oil hemp seed (*Cannabis sativa*), buckwheat (*Fagopyrum esculentum*), and quinoa (*Chenopodium quinoa*). Basic composition and various nutritional components like amino acids, sugars, minerals, and dietary fiber were determined. Nearly all the samples studied could be considered as good sources of essential amino acids, minerals and dietary fiber. The highest content of crude protein (over 30 g/100 g DW) was found in faba bean, blue lupin and rapeseed press cake. The total amount of essential amino acids (EAA) ranged from 25.8 g/16 g N in oil hemp hulls to 41.5 g/16 g N in pearled quinoa. All the samples studied have a nutritionally favorable composition with significant health benefit potential. Processing (dehulling or pearling) affected greatly to the contents of analyzed nutrients.

## Introduction

High animal protein intake has been linked to increased risk of type 2 diabetes, cardiovascular disease, colorectal cancer and early deaths, whereas plant proteins have shown significant protective effects [1, 2]. A major source of gas emissions is ruminant-based animal production. It has been estimated that in the European Union, the livestock sector accounts for between 12 and 61% of the total anthropogenic greenhouse gas emissions [3]. There is therefore an urgent need to shift towards a more plant-based diet, for both environmental and public health reasons.

Faba bean (*Vicia faba*), lupin (*Lupinus angustifolius*), rapeseed (*Brassica rapa/napus* subsp. *Oleifera*), flaxseed (*Linum usitatissimum*), oil hemp (*Cannabis sativa*), buckwheat (*Fagopyrum esculentum*), and quinoa (*Chenopodium quinoa*) may offer good alternatives to soybean, and contribute to enhancing diversity as well as the environmental and economic sustainability of local agricultural production. They are also a rich source of energy, fiber, high quality protein, macro- and micronutrients, as are good sources of bioactive non-nutrient compounds [4–7].

From a nutritional point of view, the most important aspects of a protein source are its AA and EAA content, composition and digestibility. As mentioned above, plant protein sources can also offer other nutrients and bioactive compounds.

There is still insufficient knowledge of the compositional data of faba bean, lupin, rapeseed press cake, flaxseed, oil hemp, buckwheat, and quinoa, as well as the effect of processing. The goal of the present study was to determine the various nutrients in the commercial products of these crops.

## Material and Methods

The commercial samples of whole unpearled (*n* = 1) and pearled (*n* = 1) quinoa seed, whole lupin seed (*n* = 2), rapeseed press cake (*n* = 2), dehulled buckwheat seed (*n* = 3), buckwheat bran (*n* = 1), whole faba bean (*n* = 3), hulled and grinded faba bean (*n* = 1), whole flaxseed (*n* = 3), whole oil hemp seed (*n* = 4), and hulls of oil hemp (*n* = 3) were purchased from local grocery stores or directly from farmers/producers. The sample size varied 500-1000 g. Pearled quinoa seeds were pre-processed by the farmer using abrasive milling (i.e. pearling) to remove the saponin-rich outer layer of the seed. Buckwheat samples were dehulled and rapeseed press cakes were produced from cold-pressing processes by the manufacturers. All unmilled or coarse samples were milled before analyses using a KT-120 hammer mill with a ø1mm sieve (Koneteollisuus Oy, Klaukkala, Finland). All milled samples were stored in a freezer at −20 °C before being analyzed.

### Methods

Luke laboratories (T024) comply with standard EN ISO/IEC 17025 and are accredited by the FINAS Finnish Accreditation Service (Helsinki, Finland). All methods except fiber are accredited.

The moisture content was determined by drying the samples at 105 °C overnight (17 h).

The nitrogen contents were determined with an in-house Kjeldahl method using a Kjeltec TM8400 analyzer according to the Association of Official Analytical Chemists (AOAC) method 2001.11.

The total fat content was determined using the SoxCap TM 2047 in combination with the Soxtec TM 2050 extraction system with a preparatory acid hydrolysis step and diethyl ether extraction (Foss A/B, Hillerød, Denmark) according to ISO 6492. (Animal feeding stuffs – Determination of fat content. 2011).

The total carbohydrates content was calculated with the following formula: total carbohydrates (% FW) = 100 − moisture (%) − protein content (%FW) − crude fat (%FW) − ash (%FW). The results show total carbohydrates as g/100 g FW.

The ash content was measured by burning the samples at 500 °C overnight (17 h).

The energy content was calculated with the following factors: protein 4 kcal/g, fat 9 kcal/g, and carbohydrates 4 kcal/g.

Analysis of AA tot (peptide bound and free) was done according to the Community methods of analysis for the determination of amino acids, crude oils and fats, and olaquindox in feeding stuffs and amending Directive 71/393/EEC [8]. AA tot was determinated by MassTrak UPLC (Waters, Milford, USA), using the UPLC Amino Acid Analysis Solution® application.

PER on the basis of interactions between leucine - tyrosine was calculated using the modified regression equations as described by Alsmeyer et al. [9]. PER = −0.468 + 0.454 leucine – 0.105 tyrosine where leucine and tyrosine are concentrations of these AA expressed in g/16 gN.

Sugars (fructose, glucose, maltose, raffinose, lactose and sucrose) were determined according to the method of the Nordic Committee on Food Analysis (No 148/1993) by HPLC-RI. The analytical column used was Luna NH2 150 mm*3 mm, particle size 5 μm (Phenomenex, Torrance, CA) and acetonitrile-water (75:25) was used as a mobile phase.

Total, soluble and insoluble dietary fiber was determined according to AOAC official method 991.43. Total, soluble, and insoluble dietary fiber in foods.

Mineral and trace elements were determined by ICP-OES and cadmium by ETAAS method. For the elemental composition, samples were digested in concentrated nitric acid in a block digestor (Tecator Dig40AUTO with scrubber).

### Statistical Analyses

All analyses were performed at least duplicated or triplicated. The results of the samples obtained from various sample suppliers are expressed as means and SD. One-way analysis of variance was fitted to the data. LSD was calculated for pairwise comparisons. If the difference between two samples is higher than LSD, the difference is statistically significant at 5% level. Because of imbalance of sample sizes, LSD will vary between comparisons. Median LSD was reported (=typically LSD for comparison where sample with *n* = 1 was compared to sample where *n* = 3). SAS 9.3. was used to fit the model (SAS Institute Inc., Cary, USA).

## Results and Discussion

### Basic Composition

The highest protein content was found in faba bean, blue lupin and rapeseed press cake, while quinoa samples and whole dehulled buckwheat appeared to contain lower levels (Table 1). Our mean protein results for commercial whole faba bean and blue lupin samples are in accordance with earlier studies [5, 10]. However, Multari et al. [6] obtained somewhat lower results for commercial faba bean flour and higher results for lupin flour than were found in the present study. Similar protein content of hemp and flaxseed is documented in the literature [11, 12]. Furthermore, in line with our study lower protein levels have previously been found in buckwheat and quinoa [6, 13, 14]. It seemed that protein was mostly located in the outer layer of the buckwheat and quinoa seeds, while the opposite was found in the case of oil hemp. Protein content of plant foods is influenced by cultivar, environment and grade of processing which explains variabilities between various studies [11, 12, 14].

**Table 1:** Proximate composition and dietary fiber in dry weight basis and moisture (mean ± SD/difference of average), na=not analyzed

Samples	Moisture g/100 g	Protein g/100 g DW	Fat g/100 g DW	Ash g/100 g DW	Carbohydrates g/100 g DW	Energy kJ/100 g DW	Insoluble DF g/100 g DW	Soluble DF g/100 g DW	Total DF g/100 g DW
Faba bean, whole, (*n* = 3)	11.9 ± 1.7	31.2 ± 0.4	2.1 ± 0.1	3.4 ± 0.1	63.3 ± 0.5	1685 ± 1.9	22.7 ± 2.0	2.0 ± 0.8	24.7 ± 1.8
Faba bean, hulled & grinded (*n* = 1)	9.0	35.5	2.1	4.0	58.5	1673	8.9	1.3	10.2
Lupin, whole (*n* = 2)	9.5 ± 1.3	30.5 ± 1.6	7.3 ± 0.4	3.8 ± 0.4	58.3 ± 0.8	1782 ± 0.5	42.0 ± 1.6	5.5 ± 0.4	47.5 ± 1.2
Buckwheat, whole, peeled (*n* = 3)	11.6 ± 0.8	14.8 ± 1.6	3.6 ± 0.3	2.0 ± 0.1	79.6 ± 2.0	1739 ± 4.6	6.2 ± 1.9	2.1 ± 0.5	8.3 ± 1.5
Buckwheat, bran (n = 1)	10.0	27.8	8.0	5.3	58.9	1769	5.9	2.1	8.4
Quinoa, whole, organic, (*n* = 1)	8.7	13.0	7.2	2.9	76.8	1795	6.6	3.3	9.9
Quinoa, whole, organic, pearled (*n* = 1)	9.8	4.5	1.1	<0.2	94.3	1722	4.1	1.8	5.9
Flaxseed, whole (*n* = 3)	7.1 ± 1.4	20.9 ± 1.9	46.3 ± 0.7	3.7 ± 0.1	29.1 ± 2.5	2563 ± 12	21.8 ± 1.3	8.4 ± 0.8	30.2 ± 2.2
Oil hemp seed, whole (*n* = 4)	6.7 ± 0.5	25.6 ± 0.6	34.6 ± 1.2	5.4 ± 0.3	34.4 ± 1.5	2301 ± 27	30.9 ± 1.5	2.9 ± 0.4	33.8 ± 1.9
Oil hemp, peel, (*n* = 3)	8.0 ± 1.2	14.2 ± 2.1	12.5 ± 4.2	3.5 ± 0.1	69.7 ± 6.3	1890 ± 84	na	na	na
Rapeseed press cake (*n* = 2)	10.7 ± 0.0	35.7 ± 0.1	10.4 ± 0.3	7.6 ± 0.1	46.3 ± 0.3	1779 ± 7	31.3 ± 2.7	5.1 ± 0.6	36.4 ± 3.3
Least significant difference	2.2	2.8	3.5	0.4	5.7	71	3.5	1.2	4.0

A great part of the total carbohydrates of whole lupin, hemp seed, and flaxseed as well as rapeseed press cake were constituted in fiber. Especially whole lupin contained a lot of insoluble fiber (Table 1). Whole faba bean contained 63.3 ± 0.5 g/100 g DW carbohydrates, of which 39% was fiber, also mostly insoluble. Buckwheat and quinoa contained lower levels of fiber and higher levels of other carbohydrates. Generally, our fiber results for lupin, hemp, flaxseed, buckwheat, quinoa, and faba bean are in accordance with earlier reports [5, 6, 15–17], while taking into account that partly different varieties and differently processed products were previously studied. However, the total fiber content of rapeseed press cake found in our study was much higher than the mean crude fiber content (11.6%) calculated by Lomascolo et al. [18] from various studies. One reason for this discrepancy is that crude fiber method measures basically only cellulose and insoluble lignin. The remaining carbohydrates–after excluding the amount of dietary fiber–are mainly starch [19–23].

The fat content of oil crops (hemp and flaxseed) was high (Table 1). These results are in accordance with the earlier data [15, 16]. The residual fat content of rapeseed press cake was quite high (10.4 ± 0.3 g/100 g DW) compared with earlier studies on rapeseed meal (2.5%, [18]). This difference is probably caused by the fact that the material used in the present study was a crude press cake. In line with earlier reports [5, 6, 17] the other crops contained fewer than 10 g/100 g DW of fat.

The ash content varied from <0.2 to 7.6 ± 0.1 g/100 g DW among the samples (Table 1). Processing affects the nutrient content of commercial products. For example, pearled quinoa contained less protein, ash, fat and dietary fiber than whole quinoa, buckwheat bran contained more protein, fat, and ash than whole dehulled buckwheat, and grinded faba bean contained less dietary fiber than the whole faba bean. Hemp hulls contained more carbohydrates but less protein, fat and ash than the whole seed (Table 1).

### Sugars

The content of free mono- and disaccharides in the samples is presented in Table 2. The total sugar content was highest in rapeseed press cake, where only sucrose was found. The result is in accordance with Jiang et al. [23]. In case of sucrose in faba bean, lupin and buckwheat, our results are similar to those presented earlier [22, 24, 25]. Contrary to Repo-Carrasco et al. [14], maltose was not found in the quinoa samples. Interestingly, pearling of quinoa reduced the sucrose content in particular, whereas glucose remained in the pearled seed. Further research would be needed to verify the effect of dehulling on the sucrose content.

**Table 2:** Content of free mono- and disaccharides (g/100 g DW; mean ± SD/difference of average)

Samples	Fructose	Glucose	Sucrose	Raffinose	Sum
Faba bean, whole (*n* = 3)			2.26 ± 0.18		2.26 ± 0.18
Faba bean, hulled & grinded (*n* = 1)			2.67		2.67
Lupin, whole (*n* = 2)		0.20 ± 0.01	3.00 ± 0.01	0.23 ± 0.01	3.42 ± 0.01
Buckwheat, whole, peeled (*n* = 3)		0.11	1.37 ± 0.14	0.73	1.64 ± 0.60
Buckwheat, bran (*n* = 1)		0.13	3.33		3.46
Quinoa, whole, organic (*n* = 1)	0.36	2.35	2.46		5.18
Quinoa, whole, organic pearled (*n* = 1)	0.10	2.93	0.49		3.53
Flaxseed, whole (*n* = 3)		0.23 ± 0.04	1.05 ± 0.26	0.47 ± 0.01	1.75 ± 0.29
Oil hemp seed, whole (*n* = 4)	0.46 ± 0.22	0.40 ± 0.20	1.11 ± 0.36		2.09 ± 0.12
Oil hemp, peel (*n* = 3)	0.50 ± 0.07	0.41 ± 0.13	0.37		1.03 ± 0.39
Rapeseed press cake (*n* = 2)			6.82 ± 0.03		6.82 ± 0.03
Least significant difference	0.35	0.28	0.47	0.02	0.63

In addition to the analyzed mono- and disaccharides, lupin and faba bean can contain relatively high amounts of α-galactosides, namely stachyose, verbascose and raffinose [22, 24]. Buckwheat contains also special sugars, fagopyritols, which are D-*chiro*-inositol galactosides [25].

### Amino Acid Content and Composition

It has been known for some time that plant proteins contain significant levels of non-protein nitrogen, and thus require a lower NP factor than 6.25 traditionally used in protein analysis [26]. According to Greenfield and Southgate [27], it would be more appropriate to base estimates of protein on AAdata. However, when comparing the sum of AAs in protein plants (Table 3) with the protein content gained using the Kjeldahl method with a protein conversion factor 6.25 (Table 1), it appeared that quite similar results were obtained.

**Table 3:** Amino acids of the samples on a dry weight basis (mean ± SD/difference of average)

	Samples											
Amino acids g/16 g N	Faba bean, whole (*n *= 3)	Faba bean, hulled & grinded (*n* = 1)	Lupin, whole (*n* = 2)	Buckwheat, whole, peeled (*n* = 3)	Buckwheat. bran (*n* = 1)	Quinoa, whole, organic (*n* = 1)	Quinoa, whole organic pearled (*n* = 1)	Flaxseed, whole (*n* = 3)	Oil hemp seed, whole (*n* = 3)	Oil hemp peel (*n* = 1)	Rapeseed press cake (*n* = 2)	LSD
Essential amino acids												
Lys	6.43 ± 0.19	6.16	4.73 ± 0.05	5.80 ± 0.31	5.73	5.72	6.16	4.09 ± 0.11	3.30 ± 0.09	2.57	4.86 ± 0.05	0.35
Met	0.89 ± 0.13	0.79	0.80 ± 0.01	2.14 ± 0.30	2.19	2.12	2.20	2.13 ± 0.14	2.19 ± 0.06	1.75	2.23 ± 0.03	0.32
Cys	1.18 ± 0.09	1.13	1.39 ± 0.01	2.37 ± 0.16	2.24	1.60	2.09	1.70 ± 0.06	1.40 ± 0.08	1.10	1.75 ± 0.01	0.19
Thr	3.65 ± 0.07	3.45	3.49 ± 0.04	3.82 ± 0.23	3.71	3.65	3.87	3.88 ± 0.09	3.08 ± 0.04	2.55	4.79 ± 0.06	0.23
Ile	4.10 ± 0.10	3.97	3.94 ± 0.01	3.58 ± 0.21	3.31	3.60	4.14	4.15 ± 0.04	3.37 ± 0.02	2.67	4.16 ± 0.05	0.21
Val	4.75 ± 0.11	4.56	3.95 ± 0.05	4.94 ± 0.29	4.64	4.52	5.19	5.25 ± 0.06	4.41 ± 0.04	3.62	5.52 ± 0.08	0.29
Leu	7.51 ± 0.21	7.25	6.85 ± 0.04	6.40 ± 0.40	5.90	6.15	7.02	6.05 ± 0.06	5.76 ± 0.07	4.61	7.35 ± 0.06	0.41
His	2.56 ± 0.04	2.43	2.56 ± 0.00	2.27 ± 0.17	2.26	2.52	2.13	2.09 ± 0.05	2.25 ± 0.04	1.50	2.56 ± 0.02	0.17
Phe	4.25 ± 0.11	4.07	3.86 ± 0.03	4.52 ± 0.32	4.28	3.89	4.53	4.73 ± 0.05	3.95 ± 0.05	3.41	4.20 ± 0.01	0.31
Tyr	3.67 ± 0.11	3.50	3.76 ± 0.01	3.09 ± 0.18	2.80	3.49	4.14	2.83 ± 0.08	3.04 ± 0.08	2.07	3.48 ± 0.09	0.22
Non essential amino acids												
Arg	9.73 ± 0.60	10.21	10.05 ± 0.58	10.10 ± 0.72	10.00	7.89	6.45	9.50 ± 0.14	10.51 ± 0.23	6.25	6.03 ± 0.07	0.94
Asp	11.20 ± 0.27	10.71	10.10 ± 0.02	9.73 ± 0.52	9.30	8.15	8.96	9.98 ± 0.04	9.11 ± 0.18	7.25	8.13 ± 0.08	0.55
Ser	4.98 ± 0.14	4.74	4.99 ± 0.09	4.96 ± 0.33	4.78	4.18	4.51	4.79 ± 0.05	4.39 ± 0.07	3.25	4.60 ± 0.04	0.33
Glu	16.78 ± 0.52	16.45	21.01 ± 0.60	17.72 ± 0.99	17.61	13.33	11.77	19.69 ± 0.32	14.95 ± 0.43	9.86	17.26 ± 0.10	1.16
Pro	4.22 ± 0.08	4.09	3.86 ± 0.04	3.72 ± 0.28	3.44	3.56	3.85	3.68 ± 0.02	3.48 ± 0.13	3.15	6.05 ± 0.03	0.28
Gly	4.38 ± 0.08	4.06	4.21 ± 0.01	5.99 ± 0.43	5.85	5.35	6.32	6.06 ± 0.05	4.00 ± 0.05	3.26	5.31 ± 0.06	0.39
Ala	3.97 ± 0.07	3.78	3.35 ± 0.03	4.10 ± 0.24	3.90	5.34	4.36	4.46 ± 0.01	3.66 ± 0.06	3.05	4.53 ± 0.05	0.23
AA tot	94.27 ± 2.46	91.37	92.94 ± 1.16	95.27 ± 5.95	91.95	83.82	87.69	95.09 ± 0.42	82.86 ± 1.42	61.9	92.84 ± 0.79	5.92
EAA tot	38.99 ± 0.88	37.31	35.33 ± 0.17	38.93 ± 2.52	37.05	37.26	41.48	36.91 ± 0.37	32.75 ± 0.45	25.84	40.89 ± 0.36	2.43
PER	2.53 ± 0.09	2.43	2.22 ± 0.02	2.09 ± 0.16	1.89	1.93	2.26	1.96 ± 0.04	1.81 ± 0.02	1.39	2.48 ± 0.02	0.17
AA tot (g/100 g)	29.44 ± 0.47	32.42	28.34 ± 2.51	14.07 ± 1.03	25.54	10.93	3.99	19.85 ± 1.79	21.17 ± 0.56	8.71	33.12 ± 0.37	2.51
EAA tot (g/100 g)	12.18 ± 0.13	13.24	10.77 ± 0.77	5.75 ± 0.39	10.29	4.86	1.89	7.70 ± 0.63	8.37 ± 0.24	3.63	14.59 ± 0.17	0.86

*AA* amino acids, *EAA* essential amino acids, *PER* protein efficiency ratio

Table 3 shows the AA composition and contents of the samples. The contents of individual AA, AA tot, individual EAA and EAA tot are expressed as grams of an AA per 16 g nitrogen. The AA tot and EAA tot are also expressed as g/100 g DW. Rapeseed press cake and legumes (faba bean and lupin) had the highest amounts of AA tot (g/100 g DW). However, if the oil is removed from hemp seed and flaxseed their press cakes would also be excellent sources of AA tot with levels that are comparable to those of rapeseed press cake. Also, buckwheat bran is a rich source of AA tot and EAA tot, whereas peeled buckwheat and pearled quinoa are comparatively low in AA tot and EAA tot.

AA composition is an essential factor in evaluating the nutritional quality of a dietary protein source. According to Alsmeyer et al. [9] the nutritional value should be expressed in terms of Leu and Tyr contents (PER-value) while other classifications are based on the chemical scores for 9–11 EAA. For humans, adequate quantities of Lys, Met and Try are considered necessary in food that is of high nutritional value [28]. Results in this study indicated that the PER-values ranged from 1.39 (oil hemp peel) to 2.53 (faba bean) are lower than the standard 2.7 (for casein).

As seen in Table 3 the total amount of EAAs (g/16 g N) ranged from 25.8 in oil hemp peel to 41.5 in pearled quinoa. Favier et al. [29] recommended that the EAA tot should be above 36 g/16 g N. In this study, only lupin, hemp seed, and hemp seed peel samples had lower values. The EAA levels were compared to the recommended EAA values found in whole egg protein (Lys 5.5–7.0, Met+Cys 3.5–5.7, Thr 4.0–4.7, Ile 4.0–5.4, Trp 1.0–1.7, Val 5.0–6.6, Leu 7.0–8.6, His 0–2.2, Phe + Tyr 6.0–9.3 g/16 gN) [28, 30, 31]. The species examined contained all EAAs, among which Leu and Lys were the most available. Lys level was high especially in faba bean, quinoa and buckwheat. Met+Cys levels were low in legumes (faba bean and lupin), while in other samples the amount was comparable to egg. Moreover, the concentrations of Phe and Tyr were above the recommended levels in all samples except the peeled oil hemp seeds. Overall, among the samples rapeseed press cake was the most efficient source of high quality protein, with an EAA composition comparable to those of bovine milk and egg. On the other hand, faba bean species with high Lys levels could be used for balancing the AA composition of cereal-based products typically low in Lys.

Although it is well known that peeling and pearling significantly decrease the nutritional value of staple grains (e.g. wheat) [32], the effects of these processes on the nutritional value of pseudocereals and oil seed plants have not been characterized. For that reason, one aim of the present study was to evaluate whether peeling or pearling alters the AA content and composition of quinoa, buckwheat and oil hemp seeds. The results are presented in Table 3 as AA tot and EAA tot in g/100 g DW. According to the results, AAs in pseudocereals (quinoa and buckwheat) are concentrated in the outer shell of the seed. Pearling significantly reduced the AA tot in quinoa, as well as EAA tot. In addition, AA tot and EAAs seemed to be concentrated in the bran of buckwheat. However, in oil hemp seed the opposite phenomenon was observed: oil hemp peel contained less AA tot than the whole oil hemp seed (Table 3). In summary, the results show evidently that, processing requires thorough attention regarding the raw material to preserve the protein quality in the final consumer product.

### Mineral Elements

The mineral compositions of flaxseed, buckwheat, faba bean, hemp, quinoa, lupin, and rapeseed press cake are presented in Table 4. All the seeds studied were rich sources of major minerals (Ca, K, Mg, P, S) and trace elements (Cu, Fe, Mn, Zn) compared to whole grain cereal flours [33]. Superior sources of major minerals were buckwheat bran, rapeseed press cake, hempseed, flaxseed and faba bean. Only pearled quinoa had a lower mineral content than whole grain cereals. In the pearling process about 40% of the outer layer of quinoa seed is discarded causing 50–90% decrease in mineral elements concentratios. The largest decreases were in Mn, P, Mg and K concentrations. This reduces the value of quinoa as a source of essential minerals. However, the lower mineral concentrations in pearled quinoa may be compensated for by better absorption, due to the lower phytic acid and saponin contents [7].

**Table 4:** Minerals, trace elements and cadmium of the samples on a dry weight basis (mean ± SD/difference of average)

Samples	Calcium, Ca g/100 g	Potassium, K g/100 g	Magnesium, Mg g/100 g	Phosphorus, P g/100 g	Sulfur, S g/100 g	Copper, Cu mg/100 g	Iron, Fe mg/100 g	Manganese, Mn mg/100 g	Zinc, Zn mg/100 g	Cadmium, Cd mg/100 g
Faba bean, whole, (*n* = 4)	0.103 ± 0.019	1.33 ± 0.013	0.150 ± 0.006	0.656 ± 0.103	0.212 ± 0.014	1.66 ± 0.26	2.65 ± 0.39	1.44 ± 0.15	4.85 ± 0.84	0.0016 ± 0.0005
Faba bean, hulled & grinded (*n* = 1)	0.049	1.50	0.127	0.798	0.285	2.1	3.3	1.00	7.20	0.0005
Lupin, whole, (*n* = 2)	0.310 ± 0.016	0.086 ± 0.004	0.221 ± 0.002	0.470 ± 0.429	0.287 ± 0.020	0.64 ± 0.03	2.37 ± 0.38	2.91 ± 0.70	3.40 ± 0.3	0.0022 ± 0.0001
Buckwheat, whole, peeled (*n* = 3)	0.022 ± 0.002	0.584 ± 0.014	0.270 ± 0.036	0.544 ± 0.055	0.212 ± 0.003	0.66 ± 0.11	3.51 ± 0.38	1.96 ± 0.03	3.94 ± 0.33	0.0047 ± 0.0011
Buckwheat, bran, (*n* = 1)	0.029	1.17	0.55	1.23	0.356	6.4	5.9	4.7	7.6	0.0152
Quinoa, whole, organic (*n* = 1)	0.053	1.00	0.260	0.54	0.17	0.66	2.8	4.3	3.8	0.0039
Quinoa, whole, organic, pearled, (*n* = 1)	0.021	0.29	0.066	0.11	0.077	0.25	1.5	0.46	2.4	0.0036
Flaxseed, whole, (*n* = 3)	0.233 ± 0.034	0.940 ± 0.095	0.382 ± 0.015	0.656 ± 0.103	0.212 ± 0.014	1.34 ± 0.19	3.48 ± 1.56	1.75 ± 0.24	5.17 ± 0.30	0.0269 ± 0.0073
Oil hemp seed, whole, (*n* = 4)	0.127 ± 0.007	0.921 ± 0.042	0.496 ± 0.037	1.17 ± 0.123	0.278 ± 0.010	1.89 ± 0.084	4.38 ± 0.726	10.5 ± 0.78	6.97 ± 0.38	0.0015 ± 0.0007
Oil hemp, peel, (*n* = 3)	0.166 ± 0.005	0.529 ± 0.132	0.221 ± 0.042	0.393 ± 0.135	0.141 ± 0.026	1.80 ± 0.075	3.06 ± 0.052	12.1 ± 1.27	2.92 ± 0.77	0.0015 ± 0.0006
Rapeseed press cake, (*n* = 2)	0.872 ± 0.012	1.32 ± 0.017	0.544 ± 0.007	1.39 ± 0.036	0.61 ± 0.019	0.60 ± 0.021	0.43 ± 0.142	5.60 ± 0.089	5.87 ± 0.30	0.0162 ± 0.0014
Least significant difference	0.033	0.129	0.055	0.302	0.031	0.305	1.44	1.27	1.10	0.0056

Oil hemp hulls contained 30–65% less major elements and Zn than whole seed. Cu and Mg were more evenly distributed in the whole seed. Buckwheat bran had the highest content of Cu and Fe. Oil hemp hulls and seed contained the highest amount of Mn whereas Zn was high in hulled and grinded faba bean, hempseed, rapeseed press cake and buckwheat bran (Table 4). Concentrations of major mineral and trace elements were generally in the same range as found in previous research [6, 34], but due to local conditions clear variations exist.

Cd is a toxic heavy metal. In EU regulation N:o 1881/2006, the maximum residue level for Cd in cereals is 0.1 mg/kg, and 0.2 mg/kg for bran, embryos, wheat and rice. There were no maximum residue levels for Cd in oil crops or pseudocereals. Cd content was highest in flaxseed (Table 4). Flaxseed is known to accumulate cadmium [35]. The Finnish Food Safety Authority (Evira) has recommended using only 2 spoonfuls of flaxseed per day, while bread can contain 10% of flaxseed. Buckwheat bran contained 3 times more Cd than dehulled buckwheat. Whole faba bean contained 3 times higher Cd concentrations than hulled and grinded faba bean. However, there were no large differences in Cd contents between pearled and whole quinoa and oil hemp hulls and seeds. This suggests that Cd might be relatively evenly distributed in the whole grain.

## Conclusions

In conclusion, nearly all the samples studied could be considered as good sources of protein, minerals and dietary fiber. Highest contents of protein and AA tot were determined from legumes and rapeseed press cake. The EAA composition of rapeseed press cake was the best, and comparable to the EAA compositions of bovine milk and egg. Faba bean species with high lysine levels could be used for balancing the amino acid composition of cereal-based products typically low in lysine. Dehulling and pearling greatly affected the contents of all the nutrients we analyzed.

## Abbreviations

AA: Amino acid
AA tot: Total content of amino acids
CV%: Coefficient of variation%
DW: Dry weight
EAA: Essential amino acid
EAA tot: Total content of essential amino acids
ETAAS: Electrothermal atomic absorption spectrometry
FW: Fresh weight
HPLC-RI: High performance liquid chromatography - refractive index
ICP-OES: Inductively coupled plasma - optical emission spectrometry
LSD: Least significant difference
NP factor: Nitrogen to protein conversion factor
PER: Protein efficiency ratio
SD: Standard deviation
UPLC: Ultra performance liquid chromatography

## References

[1] Chan DS, Lau R, Aune D, Vieira R, Greenwood DC, Kampman E, Norat T (2011). Red and processed meat and colorectal cancer incidence: meta-analysis of prospective studies. PLoS One.

[2] Pedersen AN, Kondrup J, Børsheim E (2013) Health effects of protein intake in healthy adults: a systematic literature review. Food Nutr Res 57. 10.3402/fnr.v57i0.21245 10.3402/fnr.v57i0.21245 PMC3730112 23908602

[3] Bellarby J, Tirado R, Leip A, Weiss F, Lesschen JP, Smith P (2013). Livestock. Greenhouse gas emissions and mitigation potential in Europe. Glob Change Biol.

[4] Friedman M (1996). Nutritional value of proteins from different food sources. A review. J Agric Food Chem.

[5] Lizarazo CI, Lampi A, Liu J, Sontag-Strohm T, Piironen V, Stoddard FL (2015). Nutritive quality and protein production from grain legumes in a boreal climate. J Sci Food Agric.

[6] Multari S, Neacsu M, Scobbie L, Cantlay L, Duncan G, Vaughan N, Stewart D, Russell WR (2016). Nutritional and phytochemical content of high-protein crops. J Agric Food Chem.

[7] Pihlanto A, Mattila PH, Mäkinen S, Pajari A-M (2017). Bioactivities in alternative protein sources and their potential health benefits. Food Funct.

[8] European Commission (1998). Commission directive 98/64/EC of 3 September 1998 establishing community methods of analysis for the determination of amino acids, crude oils and fats, and olaquindox in feeding stuffs and amending directive 71/393/EEC. Off J Eur Comm L.

[9] Alsmeyer RH, Cunningham AE, Happich ML (1974). Equations predict PER from amino acid analysis. Food Technol.

[10] Saastamoinen M, Eurola M, Hietaniemi V (2013) The chemical quality of some legumes, peas, faba beans, blue and white lupins and soybeans cultivated in Finland. J Agric Sci Technol B3:92–100. 10.17265/2161-6264/2013.02B.003

[11] Kajla P, Sharma A, Sood DR (2015). Flaxseed—a potential functional food source. J Food Sci Technol.

[12] Vonapartis E, Aubin M, Seguin P, Mustafa AF, Charron J (2015). Seed composition of ten industrial hemp cultivars approved for production in Canada. J Food Compos Anal.

[13] Giménez-Bastida JA, Zieliński H (2015). Buckwheat as a functional food and its effects on health. J Agric Food Chem.

[14] Repo-Carrasco R, Espinoza C, Jacobsen S-E (2003). Nutritional value and use of the Andean crops quinoa (*Chenopodium quinoa*) and kañiwa (*Chenopodium pallidicaule*). Food Rev Int.

[15] Callaway JC (2004). Hempseed as a nutritional resource: an overview. Euphytica.

[16] Goyal A, Sharma V, Upadhyay N, Gill S, Sihag M (2014). Flax and flaxseed oil: an ancient medicine and modern functional food. J Food Sci Technol.

[17] Nowak V, Du J, Charrondiere UR (2016). Assessment of the nutritional composition of quinoa (*Chenopodium quinoa* Willd.). Food Chem.

[18] Lomascolo A, Uzan-Boukhris E, Sigoillot J-C, Fine F (2012). Rapeseed and sunflower meal: a review on biotechnology status and challenges. Appl Microbiol Biotechnol.

[19] Giczewska A, Borowska J (2003). Nutritional value of broad bean seeds. Part 1: starch and fiber. Nahrung.

[20] Steadman KJ, Burgoon MS, Lewis BA, Edwardson SE, Obendorf RL (2001). Buckwheat seed milling fractions: description, macronutrient composition and dietary fiber. J Cereal Sci.

[21] Ruales J, Nair BM (1994). Properties of starch and dietary fiber in raw and processed quinoa (*Chenopodium quinoa*, Willd) seeds. Plant Foods Hum Nutr.

[22] Martínez-Villaluenga C, Frías J, Vidal-Valverde C (2006). Functional lupin seeds (*Lupinus albus* L. and *Lupinus luteus* L.) after extraction of α-galactosides. Food Chem.

[23] Jiang J, Wang Y, Xie T, Rong H, Li A, Fang Y, Wang Y (2015). Metabolic characteristics in meal of black rapeseed and yellow-deeded progeny of *Brassica napus-Sinapis alba* hybrids. Molecules.

[24] Frias J, Vidal-Valverde C, Kozlowska H, Gorecki R, Honke J, Hedley CL (1996). Evolution of soluble carbohydrates during the development of pea, faba bean and lupin seeds. Z Lebensm Unters Forsch.

[25] Steadman KJ, Burgoon MS, Schuster RL, Lewis BA, Edwardson SE, Obendorf RL (2000). Fagopyritols, D-*chiro*-inositol, and other soluble carbohydrates in buckwheat seed milling fractions. J Agric Food Chem.

[26] Sosulski FW, Imafidon GI (1990). Amino acid composition and nitrogen-to-protein conversion factors for animal and plant foods. J Agric Food Chem.

[27] Greenfield H, Southgate DAT (2003) Food composition data. Production, management and use, 2nd edn. Food and Agriculture Organization of the United Nations, Rome

[28] FAO/WHO/UNU (1985) Energy and Protein Requirements: Report of a joint FAO/WHO meeting. Expert consultation. Technical report series 724. World Health Organization, Geneva 3937340

[29] Favier JC, Ripert JI, Toque C, Feinberg M (1995). Repertoire general des aliments (composition tables).

[30] FAO/WHO (1991) Protein quality evaluation. Report of a joint FAO/WHO expert consultation. Rome, FAO, Food and Nutrition, pp 51

[31] Hidvégi M, Békés F, Lásztity R, Hidvégi M (1984). Mathematical modelling of protein quality from amino acid composition. Proceedings of International Association of Cereal Chemistry Symposium.

[32] Hemery Y, Rouau X, Lullien-Pellerin V, Barron C, Abecassis J (2007). Dry processes to develop wheat fractions and products with enhanced nutritional quality. J Cereal Sci.

[33] Finnish National Food Composition Database. www.fineli.fi. Accessed 31 May 2017

[34] Alvarez-Jubete L, Wijngaard H, Arendt EK, Gallagher E (2010) Polyphenol composition and *in vitro* antioxidant activity of amaranth, quinoa buckwheat and wheat as affected by sprouting and baking. Food Chem 119:770–778

[35] Saastamoinen M, Eurola M, Hietaniemi V (2016). Oil, protein, chlorophyll, cadmium and lead contents of seeds in oil and fiber flax (*Linum usitatissimum* L.) cultivars and in oil hemp (*Cannabis sativa* L.) cultivar Finola cultivated in south-western part of Finland. J Food Chem Nanotechnol.

